# Dry mouth: saliva substitutes which adsorb and modify existing salivary condition films improve oral lubrication

**DOI:** 10.1007/s00784-020-03272-x

**Published:** 2020-04-17

**Authors:** Jeroen Vinke, Hans J. Kaper, Arjan Vissink, Prashant K. Sharma

**Affiliations:** 1 Department of Biomedical Engineering, University of Groningen and University Medical Center Groningen, Antonius Deusinglaan 1, 9713 AV Groningen, The Netherlands; 2 Department of Oral Maxillofacial Surgery, University of Groningen and University Medical Center Groningen, Groningen, The Netherlands

**Keywords:** Ageing, Quality of life, Salivary lubrication, Xerostomia, Saliva substitutes, Friction, Biotribology

## Abstract

**Objectives:**

The aims of this study are to assess different saliva substitutes for their efficacy to lubricate the oral cavity, and to relate this oral lubrication to the ability of saliva substitutes to adsorb on and change the structure of the existing salivary conditioning film (SCF).

**Materials and methods:**

Quartz crystal microbalance with dissipation was used to study the capability of saliva substitutes to interact with natural SCF and the ability to change the secondary SCF (S-SCF). A tongue-enamel friction system mimicking xerostomic conditions was used to assess the relief and relief period expected from these substitutes under set circumstances.

**Results:**

Saliva Orthana spray, Biotène spray and Gum Hydral gel had an immediate effect on a SCF, increasing its structural softness. BioXtra gel, Biotène gel, Gum Hydral gel and Glandosane spray changed the S-SCF by increasing salivary protein adsorption, while others showed no sign of interaction. With respect to relief, only 2 out of the 16 saliva substitutes tested (Saliva Orthana spray and Gum Hydral gel) performed better than water. Overall, relief period correlated positively to structural softness change, whereas a positive correlation was seen between relief and mass adsorption.

**Conclusions:**

The majority of saliva substitutes did not adsorb on the SCF, thus did not enhance lubrication. Only saliva substitutes containing carrageenan, carboxymethylcellulose, pig gastric mucin, xanthan gum and carbomer performed better in enhancing oral lubrication.

**Clinical relevance:**

This objective assessment will help clinicians and patients make better choice of saliva substitutes. This study provides a scientific basis for future improvement in saliva substitutes.

## Introduction

According to the 2015 report from the Population division of the UN’s department of economics and social affairs, globally the proportion of aged as well as the overall age has never been higher and has not yet reached the peak [1]. Increasing life expectancies force countries to review and increase the retirement age, expecting the elderly to remain active, mobile and keep working for longer periods [2]. Any condition which will affect their quality of life will seriously affect their work efficiency. Xerostomia, the subjective feeling of oral dryness, is one such condition. Xerostomia is not only a symptom of increasing age [3], but it also accompanies ageing-related diseases and conditions like Sjögren’s syndrome, diabetes mellitus, side effects of several (combinations of) drugs and irradiation in the head and neck region [4, 5]. From these multiple causes, 63% of hospitalized elderly suffer from xerostomia [6].

Xerostomia is often accompanied by either decreased salivary flow or an altered composition of saliva [7, 8]. Saliva is the main substance in the mouth that provides the lubrication needed for a normal oral function like mastication, swallowing and speech, and preventing wear of mucosal tissue and dental surfaces. Therefore, the lack of saliva could have devastating effects. These effects include impeded oral functioning, a high risk of developing dental caries and oral infections and a worsened quality of life [3, 9–11].

A variety of saliva substitutes (Table 1) have been introduced to alleviate oral dryness when saliva stimulation is overall insufficient or fails to relieve xerostomia and its related complaints. Hahnel et al. and Furness et al. [12, 13] have reviewed the studies reported in literature which compare saliva substitutes. Common in both reviews is the conclusion that strong evidence is lacking for any saliva substitute to relieve dry mouth symptoms as also recognized by others [8]. This raises the question of whether the methods currently used to compare and assess saliva substitutes are valid. The methods most commonly applied include in vivo visual analogue scale, Xerostomia index, xerostomia questionnaire, dryness ranking scores [14–16] and measuring (un)stimulated whole saliva flow rate [17–20].

**Table 1:** Commercially available saliva substitutes that were used in this study. The main lubricating ingredients are indicated

Saliva substitute	Active ingredient for lubrication
Saliva Orthana (SO) spray	Porcine gastric mucin
BioXtra (BX) mouthwash	Hydroxyethyl cellulose (HEC), *Aloe vera*
BX gel-spray	HEC
BX gel	HEC, *Aloe vera*
Biotène (BT) mouthwash	HEC, *Aloe vera*
BT spray	Xanthan gum, polyethylene glycol (PEG)-hydrogenated castor oil
BT gel	HEC
Dentaid Xeros (DX) mouthwash	HEC, PEG-hydrogenated castor oil
DX spray	HEC
DX gel	HEC, *Aloe vera* 0.05%
GUM Hydral (GH) spray	PEG-hydrogenated castor oil
GH gel	Xanthan gum, carrageenan, PEG-hydrogenated castor oil
Aldiamed (ADM) spray	Carboxymethyl cellulose (CMC), *Aloe vera*
Saliva Natura (SN) spray	Plant polysaccharide
Glandosane (GDS) spray	CMC
Aequasyal (AQ) spray	Oxidized glycerol triesters
Entertainer’s secret (ES) spray	CMC, *Aloe vera*

Although measuring salivary flow and patient questionnaires can throw some light on the efficacy of a saliva substitute to relieve xerostomia, the lubricating properties of the saliva substitutes have been completely neglected. This is probably due to the absence of a reliable method to measure the lubricating properties objectively. A recently established tongue-enamel friction system has shown a relation between salivary lubricating properties and mouth feel [21]. This system can be used to compare saliva substitutes ex vivo for their extent and duration of lubricating the patient’s oral cavity, relieving the dry mouth feeling. The aim of this study was to assess saliva substitutes on their lubricity and relate it to their ability to interact with salivary conditioning films (SCF). In order to achieve this goal, quartz crystal microbalance with dissipation (QCM-D) [22, 23] and the ex vivo tongue-enamel friction system were used.

## Materials and methods

### Saliva substitutes and preparation

The selection of saliva substitutes was based on the hydrating, gelling or lubricating agents present in these substitutes, aiming for coverage of commonly applied lubricating agents available in Europe (Table 1). Some brands feature an extended product line, containing sprays, gels and/or mouthwashes. Mouthwashes and sprays were used as received. Gels were diluted to 10% in demineralized water to allow for liquid flow in QCM-D experiments.

### Human whole saliva collection and preparation

Human whole saliva was used as a control in this study. Both stimulated (SWS) and unstimulated (UWS) whole saliva were obtained from five healthy volunteers and collected and processed following standard protocols [24]. The whole saliva was collected in conformity with the relevant guidelines and regulations under the approval of the Medical Ethics Review Board of the University Medical Center Groningen (approval no. M17.217043, M09.069162 and UMCG IRB #2008109). Participants were asked not to eat or drink for 1 h before collection. Before collecting any saliva, the mouth was rinsed well with tap water.

For QCM-D experiments, reconstituted human whole saliva was used. For this, SWS of a group of 20 donors recruited at the Department of Biomedical Engineering was pooled, dialyzed and lyophilized for storage. Reconstitution was done by dissolving freeze-dried saliva in adhesion buffer (1.5 mg ml^−1^) (10% 0.5 M KCl; 0.2% 0.5 M KPi; 0.1% 0.5 M CaCl_2_ in demineralized water) [25] and stirred for 30 min at low shear rates. KPi is a solution containing 0.5 M KH_2_PO_4_ and K_2_HPO_4_. Centrifugation of reconstituted whole saliva was performed at 10,100 g at 10 °C for 5 min.

### Perturbation in structural softness of the SCF after interaction with saliva substitutes measured using QCM-D

**Fig. 1:**
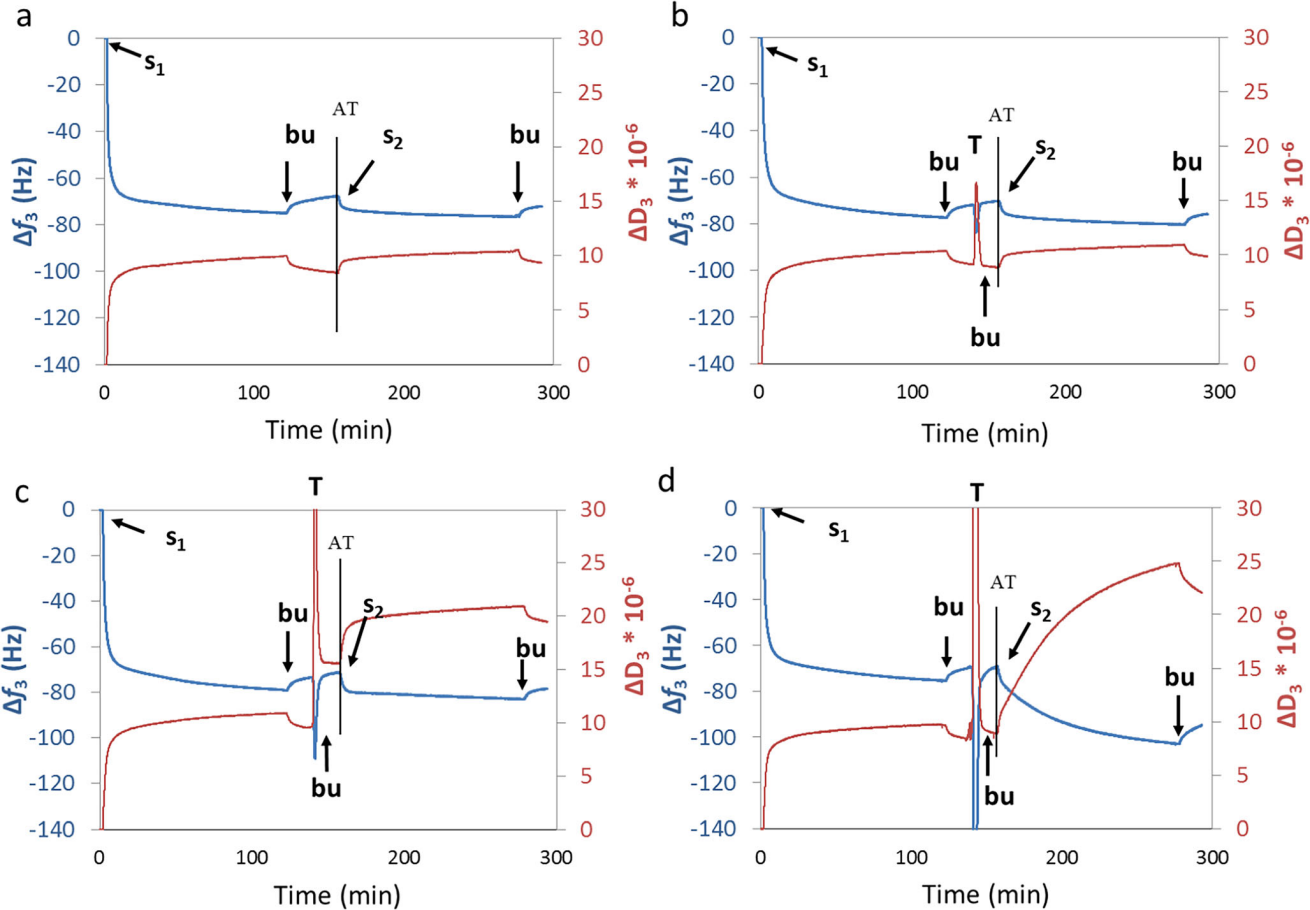
Experimental protocol used in the QCM-D to probe perturbance in SCF softness. Typical curves for frequency shift (Δ*f*) and dissipation shift (ΔD) during QCM-D experiments. Graphs are colour-matched with the axes they correspond to. Control with intermediate buffer treatment (**a**), intermediate treatment with DX gel (**b**), with SO spray (**c**) and ADM spray (**d**). Saliva was first introduced in the QCM-D chamber (*s*_1_) to create the SCF. Adsorption of saliva proteins on the substrate led to a decreased oscillating frequency with a shift of 70 Hz. At the same time, the dissipation of energy increases (**a**–**d**), meaning that the adsorbed film becomes softer. At ‘bu’, the substrate was rinsed with adhesion buffer to wash off unbound proteins (notice the frequency rise and dissipation drop due to less mass present). At ‘T’ (in **b**–**d**), the adsorbed protein layer was treated with saliva substitutes followed by rinsing with buffer ‘bu’ which resulted in different layer properties regarding Δ*f* and ΔD (**a**, control experiment; **b**, unchanged net dissipation and adsorption; **c**, increased dissipation and unchanged net adsorption; **d**, unchanged net dissipation and adsorption). At ‘*s*_2_’, reflow of saliva was done to create the S-SCF to study the interaction of new saliva proteins to the treated adsorbed layer (**a**, control experiment; **b**, unchanged net dissipation and adsorption; **c**, increased dissipation and unchanged net adsorption; **d**, increased dissipation and increased mass adsorption). Structural softness ΔD/Δ*f* of the SCF and the S-SCF were calculated ‘after treatment’ (AT) and after the final rinsing step respectively

The ability of the saliva substitute to perturb the properties of an SCF was studied using QCM-D, E4-module (Q-sense, Gothenburg, Sweden). As substrates, five MHz (sensitivity constant 17 ng cm^−2^) AT-cut gold (Au) coated quartz crystals (Jiaxing Jingkong Electronic Co., Ltd., Jiaxing, China) were used. Before experiments, the crystals were cleaned by 10 min UV/ozone treatment, then immersed in 3:1:1 mixture of ultrapure water, NH_3_ and H_2_O_2_ at 75 °C for 10 min followed by another UV/ozone treatment and placed in the QCM-D flow chamber. A protocol proposed by Veeregowda et al. [22] was used for experiments where adhesion buffer was introduced in the QCM-D chamber above the resonating crystal till constant values were reached for frequency and dissipation at all the resonating frequencies, i.e. 5 to 65 MHz. The QCM-D chamber was then perfused with reconstituted whole saliva for 2 h (s_1_), which led to the formation of an initial SCF on the substrate. This step was followed by the perfusion of a saliva substitute (T) through the system for 2 min. This step was followed by another 2 h perfusion with reconstituted whole saliva (s_2_), forming a secondary SCF (S-SCF). After each perfusion step, buffer was perfused through the chamber (bu) for 15 min for rinsing (Fig. 1). The entire experiment was performed under a constant flow of 50 μl min^−1^ provided by a peristaltic pump at 25 °C. The frequency shift (Δ*f*) and dissipation shift (ΔD) were continuously monitored in real-time. The structural softness of the adsorbed SCF after exposure to saliva substitutes, SCF after treatment (SCF AT), and the structural softness of the S-SCF were calculated to be able to assess the saliva substitutes on their activity. Structural softness is a measure of viscoelasticity of an SCF, and directly related to the lubricity of SCFs [26]. It is calculated by the ratio ΔD_3_/Δ*f*_3_ for the third overtone at the end of the buffer rinsing step, as measured by the QCM-D device.

### Lubricating properties of saliva substitutes

Lubricating (and rehydration) properties of saliva substitutes were studied by reciprocating sliding using a universal mechanical tester (CETR Inc., USA) and a newly developed tongue-enamel friction system to mimic dry mouth. In short, fresh porcine tongues (Kroon BV, Groningen, Netherlands) and polished bovine tooth enamel were used as sliding surfaces. With continuous monitoring of the coefficient of friction (COF), the enamel was rubbed over a flat spot on the porcine tongue over a distance of 10 mm in a reciprocating motion with a velocity of 4 mm s^−1^ under a constant normal load of 0.25 N. After measuring 10 cycles on dry tongue surface (stage 1 in Fig. 3a, i.e. without any lubricant), a drop of 20 μl of saliva substitute (or saliva) was added on the tongue-enamel interface, immediately causing a decrease in COF to a low value (stage 2 in Fig. 3). The ratio between COF_dry_ and COF_lubricated_ is termed as ‘relief’. Since every reciprocating cycle features a maximum COF and a median COF of which the latter is representing the overall plateau value, a relief calculated from maximum and median COF (*R*_max_ and *R*_median_ respectively) were calculated [21]. The saliva substitute was spread all over the sliding zone to keep low COF for a certain period of time after which the saliva substitute layer dried up and the COF increased (stage 3 in Fig. 3a). The duration for which the COF remained low was called the relief period (RP).

The ability of the dried saliva substitute layer to get rehydrated and to re-lubricate the oral cavity was assessed by bringing 20 μl of demineralized water at the tongue-enamel interface. A second relief, relief after rehydration (*R*_max,r_ and *R*_median,r)_ and a second relief period (RP_r_) (stage 4 in Fig. 3b) were determined.

### Statistics

The standard deviation was used for reporting the variability in the average values. For comparison between multiple saliva substitutes in the tongue-enamel friction system, a one-way analysis of variance (one-way ANOVA) was performed with a Bonferroni post-hoc test. For comparison before and after rehydration of the same sample, paired two-tailed *t* tests were performed. For comparison between multiple saliva substitutes in QCM-D, a two-way ANOVA was performed with a Bonferroni post-hoc test. Pearson’s correlation coefficient ‘*r*’ was used for correlation assessments. Statistics were done using Prism Graphpad (version 5.0).

## Results

### Perturbation in the structural softness of the SCF after exposure to saliva substitutes using QCM-D

**Fig. 2:**
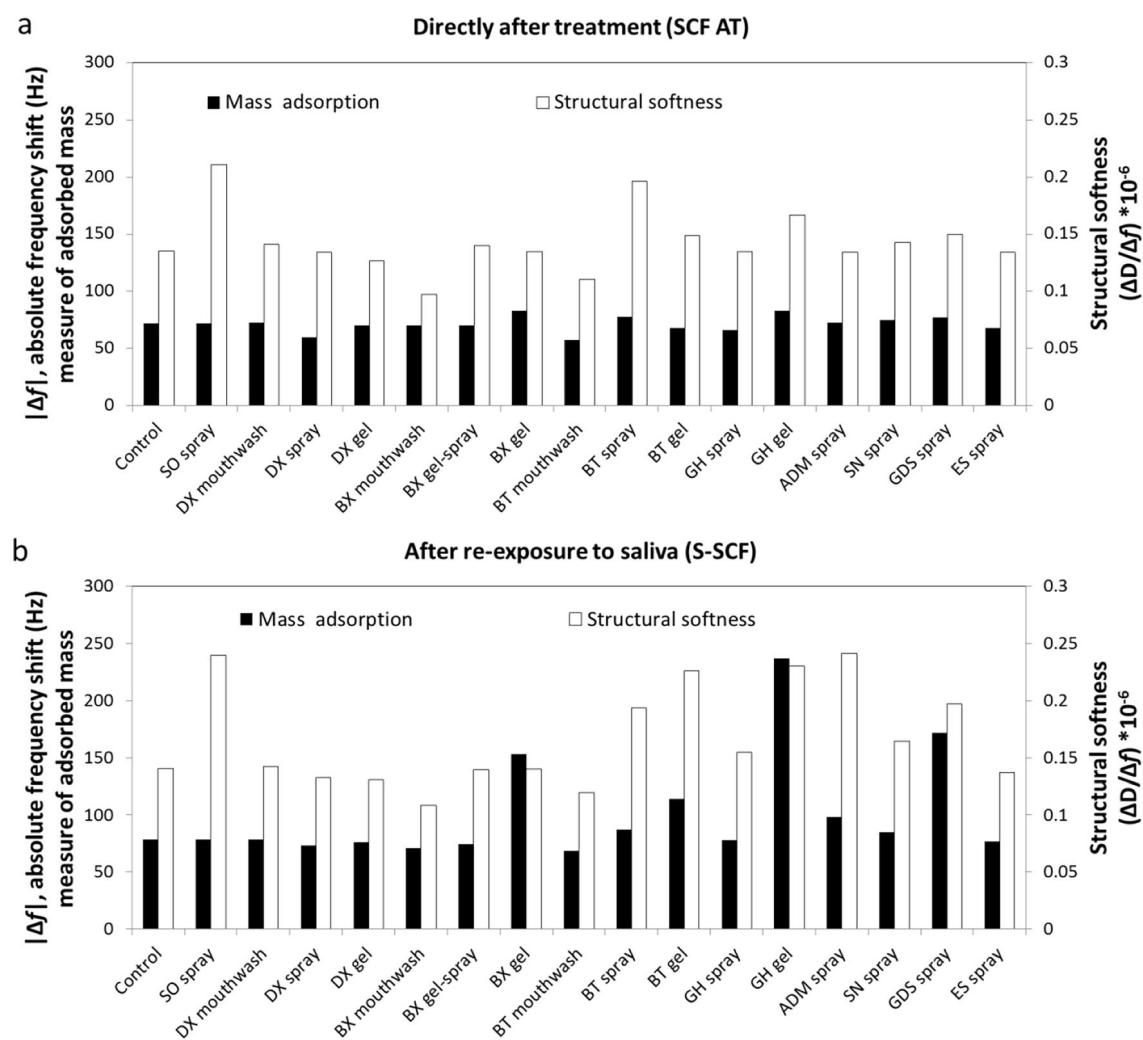
The structural softness as a measure of lubricity of the SCF film due to saliva substitute exposure using QCM-D. The mass of the adsorbed proteins on the quartz crystal measured in absolute frequency shift (|Δ*f*_3_|) and differences in the structural softness (ΔD_3_/Δ*f*_3_) of the SCF ‘after treatment’ (AT) with saliva substitutes (**a**) and after re-exposure of the SCF treated with saliva substitutes to saliva again (s-SCF: secondary SCF; **b**). The white bars represent the structural softness of the salivary conditioning film and the black bars represent the absolute frequency shift. Saliva substitutes tested: Buffer (control), Saliva Orthana (SO), Dentaid Xeros (DX), BioXtra (BX), Biotène (BT), Gum Hydral (GH), Aldiamed (ADM), Saliva Natura (SN), Glandosane (GDS), Entertainers secret (ES). All saliva substitutes except BX gel showed an increase in structural softness

Figure 1a shows the control experiment beginning with adsorption of an initial SCF for 120 min (s_1_). Hereafter, loosely bound proteins were rinsed by adhesion buffer. At this point, the structural softness (ΔD_3_/Δ*f*_3_) of the SCF was 0.14 ± 0.006 Hz^−1^ while the protein adsorption led to a Δ*f*_3_ of 71.95 ± 1.88 Hz (Fig. 2a). In Figs. 1b–d, three different saliva substitutes were applied after the first rinsing step as being a treatment (*T*), showing different outputs. The structural softness of the SCF was measured after treatment (AT), i.e. after interaction of the treatment to the SCF and after rinsing off the loosely bound molecules. Figure 1b shows the Dentaid Xeros (DX) gel not interacting well with the SCF as no change in either Δ*f* or ΔD are seen. Saliva Orthana (SO) spray interacts with the SCF by changing the softness of the layer (ΔD rises) (Fig. 1c), while ADM spray changes structural softness by changing both Δ*f* and ΔD (Fig. 1d). Figure 2a shows that after applying saliva substitutes, the structural softness (SCF AT) increased significantly for SO spray, Biotène (BT) spray and GUM hydral (GH) gel (0.21 ± 0.006, 0.20 ± 0.009 and 0.17 ± 0.009 Hz^−1^ respectively). At the same time, BioXtra (BX) mouthwash and BT mouthwash led to a decreased structural softness (0.097 ± 0.009 and 0.11 ± 0.009 Hz^−1^ respectively) (*p* < 0.05). DX mouthwash, DX spray, DX gel, BX gel-spray, BX gel, GH gel, Saliva Natura (SN) spray, Glandosane (GDS) spray and entertainer’s secret (ES) spray showed a similar structural softness as the control. The Δ*f*_3_, after applying and rinsing saliva substitutes, did change for some substitutes (SO spray and BX mouthwash), but not for the other substitutes tested, indicating that the change in ΔD_3_ was pivotal for increased structural softness changes.

QCM-D experiments featured a second cycle of 120-min exposure of the SCF to saliva (*s*_2_) followed by a second rinsing step (Fig. 1). For the control, ΔD_3_/Δ*f*_3_ of S-SCF was 0.14 ± 0.004 Hz^−1^ (Fig. 2b), which equals ΔD_3_/Δ*f*_3_ of the SCF (Fig. 2a). Comparing ΔD_3_/Δ*f*_3_ of SCF AT and S-SCF, a slight increase was found for SO spray, 0.24 ± 0.008 Hz^−1^ at the S-SCF compared with SCF AT while Δ*f*_3_ increased to 78.3 Hz. Meanwhile, a substantial increase of ΔD_3_/Δ*f*_3_ between the SCF AT and S-SCF occurred for BT gel, GH gel, ADM spray and GDS spray treatments. For BT spray, ΔD_3_/Δ*f*_3_ of S-SCF was not substantially higher than that of the SCF AT, although the Δ*f*_3_ increased by 10 Hz, i.e. from 77.8 to 87.0 Hz. The structural softness of DX mouthwash, DX spray, DX gel, BX gel-spray and ES spray show similar values for S-SCF as compared with the control. ΔD_3_/Δ*f*_3_ of BX mouthwash and BT mouthwash did not change between the SCF AT and S-SCF, but ΔD_3_/Δ*f*_3_ was lower compared with the control (0.11 and 0.12 Hz^−1^ respectively).

### Lubrication and dry mouth relief provided by saliva substitutes

**Fig. 3:**
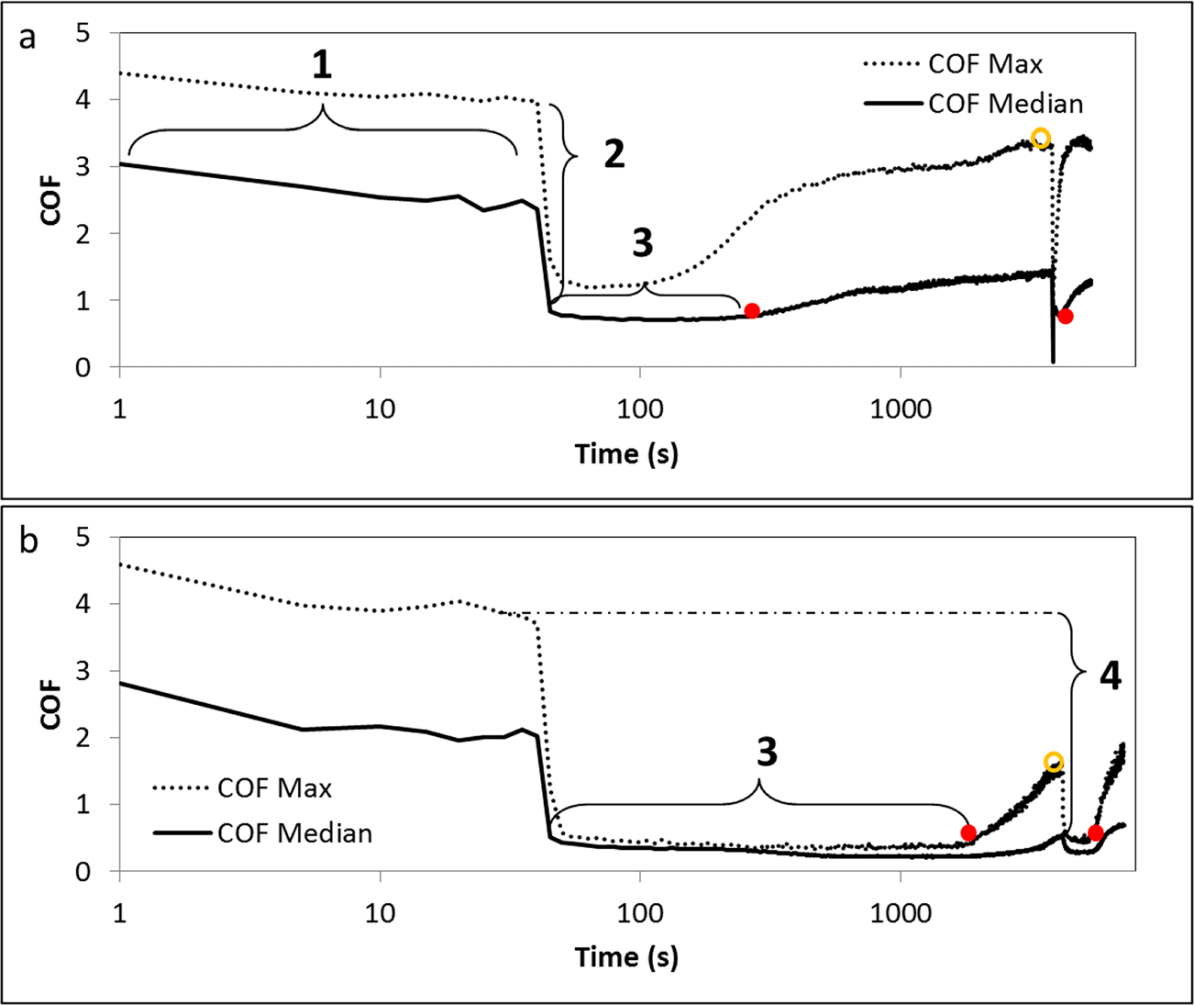
Typical output from the tongue-enamel friction system [21], shown here for two different saliva substitutes, BX gel-spray (**a**) and GH gel (**b**). Lubrication properties of saliva substitutes and their relation to the relief they provide from dry mouth. A relatively bad-performing saliva substitute with first the COF of the dry cycles (1), the relief after treatment with the saliva substitute (2) leading to a short RP (3) until the closed red dot where the slope changes clearly. After a rise of COF 25 μl of demineralized water was added causing a second drop in COF (*R*_median,r_ and *R*_max,r_) at the open orange dot leading to a secondary relief period (Rp_r_) at the second closed red dot (a). (b), the same for a relatively good-performing saliva substitute: the COF reaches a lower level compared with (a) and remains low for a longer RP. (4) Gives an indication of how the *R*_max,r_ and *R*_median,r_ were determined (also based on the dry COF). The RP_r_ shows to be relatively long in **b**

**Fig. 4:**
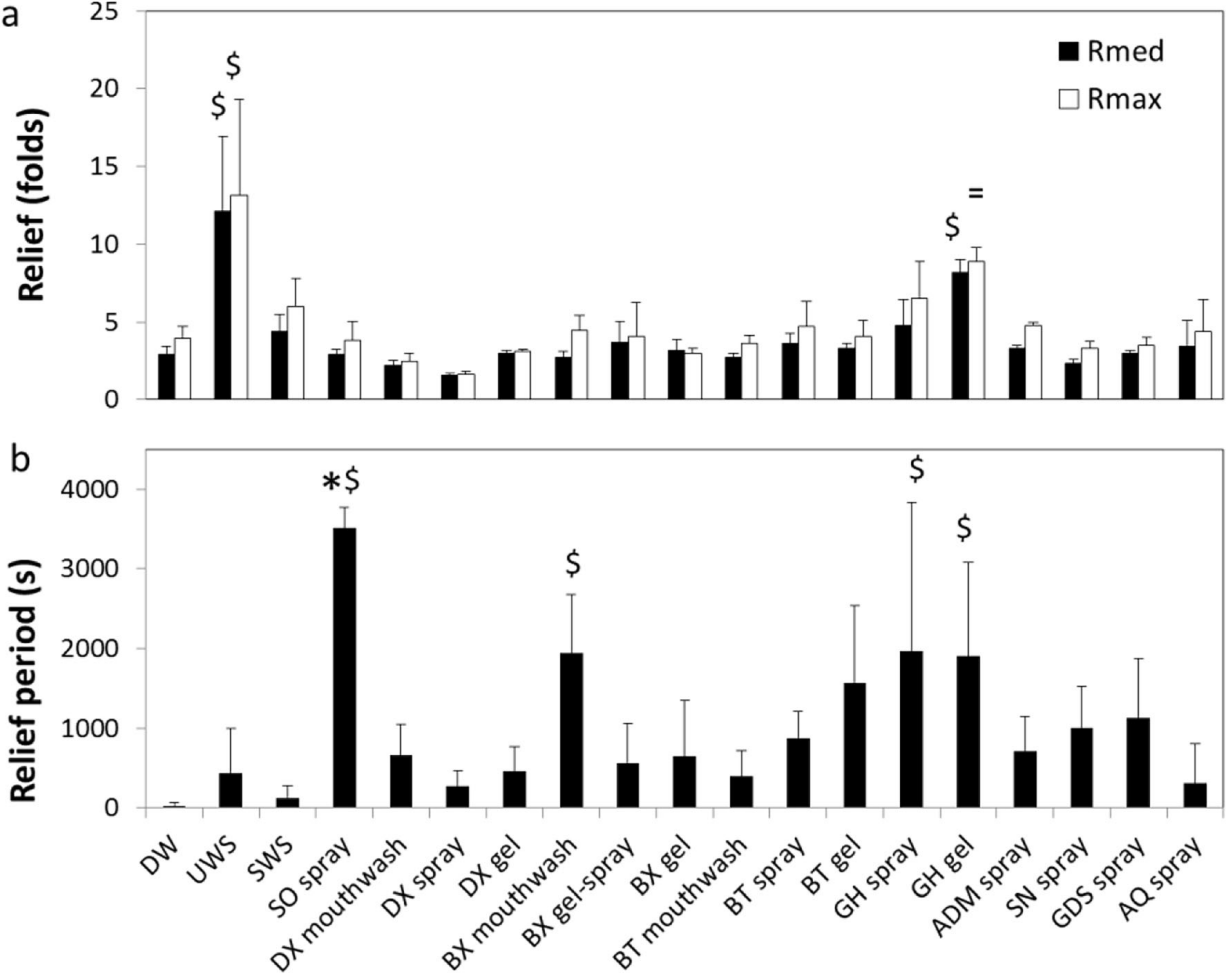
Dry mouth relief provided by saliva substitutes. Relief (**a**) and relief period (**b**) as explained in Fig. 3 obtained for different natural saliva and saliva substitutes on the tongue-enamel friction system. Relief was obtained based on both the maximum (*R*_max_) and median (*R*_median_) COF. Error bars represent standard deviations over triplicate measurements. Substances tested: demineralized water (DW), unstimulated and stimulated human whole saliva (UWS and SWS respectively), Saliva Orthana (SO), Dentaid Xeros (DX), BioXtra (BX), Biotène (BT), Gum Hydral (GH), Aldiamed (ADM), Saliva Natura (SN), Glandosane (GDS), Aequasyal (AQ). In (**a**), all tested agents were significantly different (*p* < 0.05) to UWS in both *R*_max_ and *R*_med_ except GH gel, depicted by ^=^. ^$^ depicts significant differences compared with DW (*p* < 0.05). * Shows significant difference compared with UWS in Relief Period. Data for DW, SWS, and UWS was taken from [21]

Figure 3 shows examples of typical output from the tongue-enamel friction measurements on the universal mechanical tester for both a relatively bad performing (BX gel-spray) and a relatively good performing (GH gel) saliva substitute. Figure 3a shows that the COF drops to just below 1, whereas in Fig. 3b the COF drops to below 0.5 for the median COF. The latter one led to a higher Relief. The RP in Fig. 3a is about 200 s, whereas the RP in Fig. 3b is around 1900 s, which depicts a clear difference in RP provided by the two saliva substitutes. After rehydration (open dot) a secondary relief and relief period (until next closed dot) have been visualized (*R*_median,r_, *R*_max,r_ and RP_r_). For these two, and all other tested saliva substitutes, both the *R*_max_ and *R*_median_ are displayed in Fig. 4a. Demineralized water (DW) showed a *R*_max_ and *R*_median_ of 3.9 ± 0.7 and 2.9 ± 0.5, respectively. UWS showed significantly (*p* < 0.01) higher relief of 12.1 ± 4.8 and 13.1 ± 6.2, which is about 3.3–4.2 times higher than DW. All the other tested natural saliva and saliva substitutes did not show any significant difference in relief as compared with water. For SWS, the *R*_max_ and *R*_median_ were 6.0 ± 1.8 and 4.3 ± 1.1, respectively. For the saliva substitutes, DX spray revealed the lowest relief of 1.65 ± 0.2 and 1.6 ± 0.2 for *R*_max_ and *R*_median_, whereas GH gel displayed the highest relief with *R*_max_ and *R*_median_ of 9 ± 1 and 8.5 ± 0.8 respectively. GH gel was the only saliva substitute that did not perform significantly (*p* < 0.05) worse than UWS in both parameters. The other saliva substitutes showed *R*_max_ ranging between 2.5 ± 0.6 (DX mouthwash) to 6.5 ± 2.5 (GH spray) and for *R*_median_ between 2.2 ± 0.4 (DX mouthwash) and 4.7 ± 1.7 (GH mouthwash). Relief determined using median COF per cycle correlated very well with the relief determined using the maximum COF per cycle (*r* = 0.94 in Fig. 6a).

Human whole saliva showed a relief period of 439 ± 561 s and 125 ± 155 s for UWS and SWS respectively, which was not significantly different from water (28 ± 44 s). SO spray showed a relief period from 3507 ± 259 s which was significantly higher than all other lubricants (*p* < 0.05) except four other saliva substitutes which showed a high mean in relief period accompanied by a high standard deviation (GH gel and spray, BT gel and BX mouthwash) (Fig. 4b). Despite the high mean values of relief period, these saliva substitutes showed no significant differences from water. Regardless of the high standard deviation in the relief period, Fig. 6b illustrates a fairly strong correlation between relief and relief period, (*r* = 0.63 and 0.76 for median and maximum respectively), although beyond the relief period of 2000 s, we only observed an increase in relief.

### Dry mouth relief provided by saliva substitutes upon rehydration with water

**Fig. 5:**
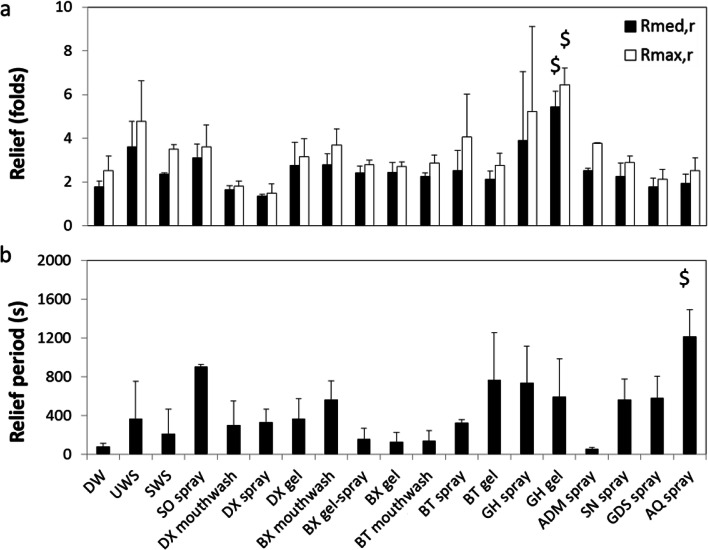
Dry mouth relief provided by saliva substitutes upon rehydration with water. Relief (**a**) and relief period (**b**) after rehydration obtained for different natural saliva and saliva substitutes on the tongue-enamel friction system. Relief after rehydration was obtained based on both the maximum (*R*_max,r_) and median (*R*_median,r_) COF. Error bars represent standard deviations over triplicate measurements. Substances tested: demineralized water (DW), unstimulated and stimulated human whole saliva (UWS and SWS respectively), Saliva Orthana (SO), Dentaid Xeros (DX), BioXtra (BX), Biotène (BT), Gum Hydral (GH), Aldiamed (ADM), Saliva Natura (SN), Glandosane (GDS), Aequasyal (AQ). $ shows significant differences (*p* < 0.05) to DW

**Fig. 6:**
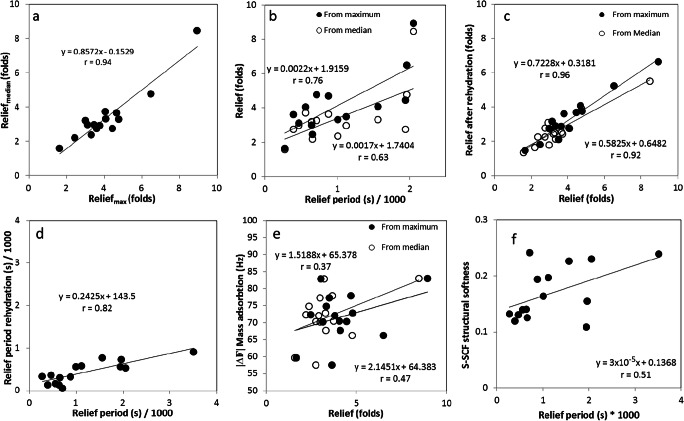
Lubrication depends on the perturbation to the structural softness of the salivary conditioning films by saliva substitutes. Correlation between different lubricating properties of saliva substitutes and with the perturbation caused to the SCF; Relief_max_ vs. Relief_median_ (**a**), relief period vs. relief (**b**), relief before and after rehydration (**c**), relief period before and after rehydration (**d**), relief vs. protein adsorption after treatment with saliva substitutes (**e**) and relief period vs. structural softness of the S-SCF after second perfusion with saliva (**f**). For every graph, the Pearson’s correlation coefficient ‘*r*’ has been visualized

Figure 5a shows the relief after rehydration (*R*_med,r_ and *R*_max,r_) of the once dried-up layer of saliva substitutes. This was done to study the possibility of the reuse of the adsorbed layer of the saliva substitute in the patient’s mouth simply with the help of water. The figure shows that the relief after rehydration is highly comparable for most saliva substitutes. DW had *R*_med,r_ of 1.79 ± 0.265 which was significantly lower than the first time (*p* < 0.05). SWS, the BT product family, GH gel and GDS had a significantly worse *R*_med,r_ compared with *R*_med_. The remainder of the saliva substitutes did not show any differences between *R*_med_ and *R*_med,r_. Overall GH gel is the only saliva substitute that performed significantly better than water in both *R*_med,r_ and *R*_max,r_. Altogether, there is a strong correlation between the initial relief and the relief after rehydration (*r* = 0.96 and *r* = 0.92 for *R*_max_ and *R*_med_ respectively) (Fig. 6c). In relief period (Fig. 5b) after rehydration (RP_r_), AQ spray revealed a significant longer relief period than demineralized water (but not to UWS). No significant differences were found in relief period duration between initial RP and RP_r_ except for SO spray, which performed significantly worse than the first time. Altogether, the overall RP and RP_r_ correlate well (*r* = 0.82) (Fig. 6d). RP_r_ is only about one-fourth of the initial relief period.

### Correlation between structural and lubrication parameters

Besides the correlation of tongue-enamel parameters, the parameters between the methods have been analysed. Figure 6e shows a fair correlation with *r* = 0.47 and 0.37 for *R*_median_ and *R*_max,_ respectively and adsorbed protein mass (Δ*f*_3_) on the SCF, while the relief period correlates better with the structural softness (Fig. 6f) of the SCF (*r* = 0.51).

## Discussion

In this study, we have assessed the ability of various saliva substitutes to lubricate, as a means to provide relief to xerostomia patients, in relation to their capacity to interact with the existing SCF by changing its structural softness.

The lubricating properties were measured using a tongue-enamel friction system [21], where the relief and relief period were calculated based on median and maximum COF per reciprocating cycle. Since relief determined using median COF per cycle correlated very well with the relief determined using the maximum COF per cycle, either of them may be used for further comparison of the lubricating properties of saliva substitutes and natural saliva. The fairly strong correlation between relief and relief period (Fig. 6b) exists up to about 2000 s of relief period and this might be explained by the nature of the two parameters. Both relief and relief period provided by human whole saliva and saliva substitutes are dependent on their chemical composition and presence of specialized lubricating and water holding molecules.

Mechanical stimulation of the salivary flow increases the parotid contribution to the total saliva [27]. The result is that UWS contains a higher contribution from the thick, mucin-containing, submandibular and sublingual saliva than SWS. The thicker composition of UWS provides three times higher relief as compared with SWS (Fig. 4 and [21, 28]). All of the saliva substitutes but GH gel showed the relief which was significantly lower than UWS but similar to SWS and water (Fig. 4a). This indicates that with respect to relief, saliva substitutes do not perform any better than water and some even worse. It suggests that most saliva substitutes lack good lubricating properties. This corroborates the concluding statement from Furness et al. [12], i.e. “There is no strong evidence from this review that any topical therapy is effective in relieving the symptoms of dry mouth.”

Relief period is highly dependent on ambient air humidity and temperature. Ambient air humidity and temperature were hard to control in our setup, which might have influenced the drying rate of the saliva and saliva substitutes ex vivo. One way in which human saliva lubricates the oral cavity is via salivary mucin MUC5B, which adsorbs on the mucosa in both healthy and dry mouth patients [29]. In a highly humid oral cavity, mucins enable lubrication by trapping water molecules [29, 30]. In a less humid environment, i.e. in air like in our experiments, much less water can be retained by the mucins leading to easier drying of the mucin layer. This probably caused high standard deviations in some measurements, resulting in insignificant differences. A larger sample size could have been more conclusive regarding the relief period; however, our results provide an overview of the efficacy of saliva substitutes in general. With respect to relief period, one saliva substitute relieved the dry mouth for a much longer period of time than UWS, i.e. SO spray. Some others (BX mouthwash, GH spray, and GH gel) provided relief significantly longer than DW, while the remainder performed no better than DW, i.e. < 300 s (Fig. 4b). A short relief period provided by UWS and SWS might be explained by the differences in humidity between the ex vivo system used and the oral environment. In the ex vivo setup, the mucin layer is more easily dried out compared with the oral cavity. However, when the dried salivary layer was rehydrated, the functionality of the UWS was restored for a longer period of time (Fig. 5b). This suggests that even after drying the mucins are still able to reabsorb and retain water upon rehydration.

With respect to RP_r_, the outstanding results of AQ spray could be due to the oxidized glycerol triesters. It is likely that these lipid molecules will form a slippery emulsion of oil and water. SO spray had good RP_r_ here as well (~ 900 s), which shows the capacity of the porcine gastric mucin to reabsorb and bind water molecules for a longer period of time.

Notable is that the saliva substitutes which show better performance in the tongue-enamel system (Fig. 4) also show changes in the frequency shift and structural softness of SCF determined by the QCM-D (Figs. 2, 6e, f). This indicates that these saliva substitutes contain molecules that actively adsorb on the SCF and by doing so increase the Relief. We presume that mucin (from SO spray), carboxymethyl cellulose (CMC) (from ADM and GDS sprays), carrageenan and xanthan (from GH gel) and carbomer (from BX gel and BT gel) adsorb on the SCF and help increase the Relief. The relief period better correlates with the SCF softness (Fig. 6f). Saliva substitutes, which upon adsorption increase the SCF structural softness, increase the relief period. An increase in structural softness of the salivary conditioning films has been shown to cause a decrease in the COF of SCF in vitro [22]. These results suggest that if a saliva substitute is able to interact with the SCF, it will provide better lubricating properties.

The makers of most of the saliva substitutes only mention the molecules they have used in the formulation but do not mention the amounts (% *w*/*v*) used, which makes it difficult to relate the changes caused by them to the SCF and its lubricity. Also, synergistic effects between different constituents cannot be ruled out. Only one out of eight saliva substitutes containing hydroxyethyl cellulose (HEC) (BT gel) increased the relief period and increased the structural softness of the S-SCF. A major difference in BT gel composition and the other saliva substitutes containing HEC is that it also contains the polymer carbomer (poly(acrylic acid)), which has been shown to have mucoadhesive properties [31, 32]. However, the other saliva substitute containing only carbomer (BX gel) only increased the mass adsorption but did not increase structural softness after the treatment. This strongly indicates that the synergistic effect of HEC and carbomer, i.e. HEC containing saliva substitutes, seems only effective when carbomer is added to the substitute. HEC on its own is highly soluble in water [33] and increases viscosity.

Saliva substitutes containing CMC (ADM spray, GDS spray) changed the structural softness of the SCF, while not containing a lubrication-inducing polymer like carbomer. CMC is mucoadhesive [34] and our QCM-D results suggest that there is a strong interaction between CMC and the SCF. GDS spray caused a frequency shift of − 172 Hz, which was the highest seen after GH gel, while the S-SCF with ADM shows the highest structural softness.

Carrageenan is able to form a polymer/mucus gel [35], which could explain the differences between GH gel and GH spray. The interaction of GH gel on the structural softness of the SCF reveals a high value (~ 0.23) and very high mass adsorption (3 times control), whereas GH spray was highly comparable with the control. GH gel contains only four extra ingredients as compared with GH spray, i.e. two dyes (tartrazine and brilliant blue FCF), xanthan gum and carrageenan powder. For GH gel, it was proposed that carrageenan was the main ingredient resolving muco-adhesiveness and lubricating properties; however, xanthan gum is also likely to interact with an SCF. From in vivo intraoral bio-adhesion tests, xanthan remained for 2.5 h on four different sites in the oral cavity [36]. Furthermore, xanthan gum is generally used as a thickening agent in food and the cosmetic industry, revealing high viscosity at low concentrations [37] and has antifungal properties [38]. Other common ingredients such as glycerine, xylitol, sorbitol, starch hydrolysates and propylene or butylene glycols seem not to bear any major lubricating or SCF adapting properties. Polyethylene glycol (PEG)-hydrogenated castor oils are more frequently used as solubilizers, and to our knowledge, do not have lubricating properties [39]. Some saliva substitutes only increase viscosity instead of lubrication, while those terms are not exchangeable [13, 40].

Upon reflow of saliva on the saliva substitute-exposed SCF, we observe that salivary proteins adsorb and form an S-SCF with a different structural softness (Fig. 2b) compared with before (Fig. 2a). This implies that the components of saliva substitutes (SO spray, BT gel, GH gel, ADM spray, SN spray and GDS spray), which had adsorbed to the SCF, change the way the SCF interacts with the salivary components and thus modify the structural softness of S-SCF as compared with control. Components like mucin, CMC, xanthan gum, carrageenan, carbomer or their combinations help change the S-SCF structural softness. This increase in softness would imply that they would further enhance the ability of the S-SCF to retain water and thus increase the relief period (Fig. 6f), although this remains to be experimentally confirmed.

## Conclusions

Saliva substitutes which caused mass adsorption to SCF increased the relief, whereas the ones which increased the structural softness tend to increase the relief period. Overall, only those saliva substitutes which perturbed the existing SCF were able to enhance the lubricating and water holding capacity of the SCF and hence provide relief against dry mouth. So altogether, the presence of constituents like carrageenan, CMC, xanthan gum, carbomer and porcine gastric mucin in saliva substitute formulations were found important for their performance. Thus, there is a great need to rethink the strategy for new saliva substitute formulations. They need to contain ingredients that specifically adsorb to the existing salivary conditioning films in the patient’s oral cavity and drastically enhance the layer softness.

## Abbreviations

ADM: Aldiamed
AT: After treatment
AQ: Aequasyal
BT: Biotène
BX: BioXtra
CMC: Carboxymethyl cellulose
COF: Coefficient of friction
DW: Demineralized water
DX: Dentaid Xeros
E4: A specific type of module for QCM-D
ES: Entertainer’s secret
GDS: Glandosane
GH: GUM hydral
HEC: Hydroxyethyl cellulose
PEG: Polyethylene glycol
QCM-D: Quartz crystal microbalance with disspation
*R*_medianr_: Relief calculated from median COF
*R*_median,r_: *R*_median_ after rehydration
*R*_max_: Relief calculated from maximum COF
*R*_max,r_: *R*_max_ after rehydration
RP: Relief period
RP_r_: RP after rehydration
S-SCF: Secondary salivary conditioning film
SCF: Salivary conditioning film
SCF AT: Salivary conditioning film after treatment
SN: Saliva Natura
SO: Saliva Orthana
SWS: Stimulated whole saliva
T: Treatment
UWS: Unstimulated whole saliva
